# When Loneliness Leads to Help-Seeking: The Role of Perceived Transactive Memory System and Work Meaningfulness

**DOI:** 10.3390/bs15111506

**Published:** 2025-11-06

**Authors:** Sujin Lee, Woonki Hong

**Affiliations:** School of Business Administration, Konkuk University, Seoul 05029, Republic of Korea

**Keywords:** loneliness, help-seeking, conservation of resources theory, theory of planned behavior, work meaningfulness, transactive memory system

## Abstract

This study investigates the conditions under which workplace loneliness influences employees’ help-seeking behavior. Drawing on the conservation of resources theory and the theory of planned behavior, we propose that loneliness does not uniformly discourage interpersonal engagement but can motivate help-seeking under certain circumstances. Using survey data from 260 full-time Korean employees, we find that workplace loneliness is positively associated with help-seeking when employees perceive high levels of transactive memory systems or work meaningfulness. These moderating effects suggest that the negative impact of loneliness on help-seeking can be attenuated or reversed when key contextual and motivational resources are present. We discuss the implications of these findings for understanding workplace loneliness as a potentially adaptive response rather than solely a detrimental experience.

## 1. Introduction

Loneliness is increasingly recognized as a “modern epidemic” that affects individuals across all age groups and social backgrounds, with a growing body of research highlighting its serious consequences for both physical and mental health (Masi et al., 2011; Ng, 2024). While traditionally considered a personal or societal issue, loneliness has become an important organizational challenge, particularly in the aftermath of the COVID-19 pandemic (Lowman et al., 2024).

Workplace loneliness, defined as the subjective experience of lacking desired social connections at work (Hawkley & Cacioppo, 2010; Wright et al., 2007), has been linked to a variety of negative outcomes, including reduced psychological well-being, lower job satisfaction, and diminished performance (Blomqvist et al., 2023; Bryan et al., 2023; Ozcelik & Barsade, 2018). More importantly, workplace loneliness can hinder help-seeking behavior, which serves as an essential component of organizational learning and collaboration (e.g., Ozcelik & Barsade, 2018). This is particularly concerning because help-seeking enables employees to acquire knowledge, solve problems, and build social connections—outcomes that could potentially alleviate loneliness itself. Yet despite its potential benefits, lonely employees tend to avoid help-seeking in an effort to protect and conserve their already depleted social resources.

Existing research, grounded in conservation of resource (COR) theory, suggests that lonely employees avoid help-seeking to protect their limited social and emotional resources (Halbesleben et al., 2014; Hobfoll, 1989). This resource-protection perspective has been valuable in documenting the negative consequences of loneliness. However, recent work calls for a more nuanced understanding of loneliness, noting that it may also motivate individuals to rebuild social ties when appropriate conditions are present (J. T. Cacioppo et al., 2014; Gabriel et al., 2021; McCarthy et al., 2025). This raises an important question: under what conditions might workplace loneliness lead to constructive rather than avoidant responses?

**Figure 1:**
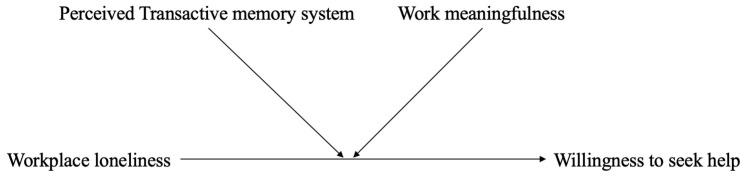
Conceptual Model of the Moderating Effects of TMS and Work Meaningfulness. Note: This model illustrates that the relationship between workplace loneliness and willingness to seek help is contingent on perceived TMS and work meaningfulness.

To address this gap, we integrate the COR theory and the theory of planned behavior (TPB; Ajzen, 1991) and examine two workplace conditions (i.e., transactive memory system and perceived work meaningfulness) that may alter how lonely employees evaluate and engage in help-seeking. Transactive memory systems reflect employees’ awareness of who knows what within their team, providing cognitive clarity about where and how to seek help (Lewis, 2003). Work meaningfulness captures the extent to which employees view their work as purposeful and significant, offering intrinsic motivation to pursue task-related goals despite social discomfort (Rosso et al., 2010). These conditions represent distinct yet complementary pathways that align with key components of TPB, with TMS enhancing perceived behavioral control by reducing uncertainty about the feasibility of help-seeking and work meaningfulness strengthening positive attitudes toward its value. Our study provides a more context-sensitive understanding of workplace loneliness. The proposed research model is presented in Figure 1.

## 2. Literature Review and Hypothesis Development

### 2.1. Workplace Loneliness and Help-Seeking

According to COR theory, individuals are motivated to acquire, retain, and protect resources that are essential for managing demands and achieving goals (Hobfoll, 1989). These resources may include emotional energy, social support, and interpersonal trust. When individuals perceive that these resources are threatened or depleted, they shift from a resource-investment to a resource-protection orientation aiming at preventing further loss (Halbesleben et al., 2014).

Within this framework, workplace loneliness reflects a condition of resource depletion—a state in which employees experience a shortage of key social and emotional resources necessary to perform and interact effectively at work (Wright et al., 2007). The lack of social connections drains social resources such as trust, social support, and belonging, and heightens sensitivity to social threat, including fear of rejection or negative evaluation. These experiences are not merely emotional but represent a cognitive and motivational shift toward self-protection and withdrawal, which can constrain employees’ willingness to engage in proactive social behaviors (Lam & Lau, 2012; Ozcelik & Barsade, 2018).

For example, when employees face relational deficiency, they become more vigilant to social risks and adopt a resource-protection orientation to avoid further loss (Fan et al., 2025; Firoz & Chaudhary, 2022). Although help-seeking behavior is generally recognized as a proactive and goal-oriented behavior that facilitates performance, learning, and collaboration (Bamberger, 2009; van der Rijt et al., 2013), it also requires emotional and social investment. Seeking help often involves revealing one’s own limitations, the risk of rejection or negative judgment, and threatening one’s self-image or social standing (Lee, 2002). From a COR perspective, these costs are additional expenditures of emotional and social resources that lonely employees—already under resource strain—are motivated to avoid (J. T. Cacioppo & Hawkley, 2009; Ozcelik & Barsade, 2018). Supporting this resource-protection mechanism, research has shown that emotional exhaustion mediates the link between workplace loneliness and withdrawal behaviors, which serve as defensive tactics that provide relief to resource-depleted employees (Xin et al., 2024). In contrast, when social-connection resources are sufficient, employees may anticipate more supportive responses from peers, lowering perceived risk in disclosing limitations. As a result, help-seeking is more likely to be construed as resource expansion rather than a resource-protection pathway (Fan et al., 2025).

**Hypothesis** **1.** *Workplace loneliness is negatively associated with employees’ help-seeking behavior.*

### 2.2. Moderating Role of Transactive Memory System

While workplace loneliness can inhibit employees’ willingness to seek help, this reluctance is not determined solely by feelings of isolation. Rather, it may be shaped by how employees perceive their team’s social and informational structure. We suggest that a well-developed TMS can serve as a contextual moderator that weakens or even reverses the negative effects of loneliness.

TMS refers to a shared cognitive system within a team that is built on agreement about “who knows what” and “whom to approach for specific knowledge” (Hollingshead, 2001; Lewis, 2003; Wegner, 1987). Rather than requiring each team member to hold all necessary information individually, a TMS allows individuals to perceive that expertise is distributed and accessible within the team. Although TMS was originally conceptualized as a team-level construct, recent studies have demonstrated that individual employees form subjective perceptions of how team expertise is structured and coordinated. These individual-level perception of TMS have been linked to important personal outcomes such as knowledge sharing and career resilience (Jarvenpaa & Majchrzak, 2008; Liu et al., 2019).

In this study, we adopt an individual-centric perspective and conceptualize TMS as an employee’s perception that expertise within the team is well-organized, trustworthy, and accessible. This perspective encompasses the three interrelated dimensions identified by Lewis (2003): recognizing differentiated expertise (specialization), trusting the quality and reliability of others’ knowledge (credibility), and understanding how to effectively access and integrate that distributed expertise (coordination). A strong perceived TMS provides not just a map of knowledge, but also a sense of predictability and psychological safety in navigating help-seeking interactions. By reducing uncertainty about how to seek help and increasing confidence that the attempt will be successful, TMS enhances an employee’s perceived behavioral control over the act of help-seeking (Ajzen, 1991).

From the TPB, behavioral intention is shaped by one’s attitude toward the behavior, perceived social norms, and perceived behavioral control (Ajzen, 1991). Building on this framework, we propose that when employees perceive their team as having a well-developed TMS, this perception can help offset the negative effects of workplace loneliness by positively shaping these components, allowing lonely employees to reinterpret help-seeking not as a potential source of resource loss, but as a viable path toward resource gain.

First, when perceived TMS is high, lonely employees are more likely to interpret help-seeking as a task-focused and normatively accepted behavior rather than a sign of weakness or interpersonal risk (Lee, 2002). The shared understanding of who knows what—reflected in statements like “That’s something A knows best” or “You should check with B on that”—can reshape how lonely employees interpret help-seeking. These cues reduce the social threat associated with asking for help and foster more positive attitudes toward asking for help (Hong & Gajendran, 2018).

Second, perceived TMS enhance subjective norms by making lonely employees feel that help-seeking is supported and expected by others (Lewis, 2004). In teams where asking questions and offering support are common and reinforced by team norms, lonely individuals are more likely to perceive social backing for help-seeking, which helps reduce fears of negative judgment or rejection (e.g., Morrison et al., 2015). Additionally, perceived TMS reduces interpersonal uncertainty by clarifying norms of communication and shared expectations for interaction (Moreland & Myaskovsky, 2000).

Third, high perceived TMS may buffer the negative impact of workplace loneliness on perceived behavioral control by providing clear cues about who holds what knowledge and how to access it. For instance, in teams where norms such as “A is the go-to person for design issues” are well established, even lonely employees, who might otherwise feel uncertain or hesitant, can perceive their help-seeking efforts as appropriate and likely to succeed (Moreland & Myaskovsky, 2000). This enhances their confidence in initiating support-seeking behavior, thereby reducing the inhibitory effects of loneliness.

Taken together, a strong perceived TMS may help lonely employees reinterpret help-seeking as a resource-gaining behavior rather than a risky investment. In such context, the negative effects of loneliness are attenuated or even reversed, as employees feel empowered to seek assistance in ways that support their goals and maintain their professional standing. In contrast, when TMS is weak or unclear, lonely employees are more likely to perceive help-seeking as uncertain and threatening, reinforcing a resource-conserving orientation that suppresses help-seeking behavior.

**Hypothesis** **2.** *The negative relationship between workplace loneliness and help-seeking behavior is moderated by the perceived TMS, such that this relationship is attenuated when perceived TMS is high but becomes stronger when perceived TMS is low.*

### 2.3. Moderating Role of Work Meaningfulness

While employees’ perception of team knowledge structure such as TMS can buffer the negative effects of workplace loneliness, employees’ motivational states may also play a critical role in shaping their help-seeking. In particular, the degree to which employees find their work meaningful can serve as an important psychological moderator that influences whether loneliness leads to withdrawal or encourage help-seeking behavior.

Work meaningfulness refers to the extent to which individuals perceive their work as purposeful, significant, and aligned with their core values and identity (Rosso et al., 2010; Steger et al., 2012). It has been shown to foster motivation, engagement, and persistence—even under stressful or socially adverse conditions. When employees perceive their work as meaningful, they are more likely to maintain intrinsic motivation and persist in goal-directed behavior, even in challenging circumstances (Lips-Wiersma et al., 2023; Steger et al., 2012).

Drawing on the TPB (Ajzen, 1991, 2002), we suggest that work meaningfulness moderates the negative effect of workplace loneliness on help-seeking behavior. Specifically, employees who perceive their work as meaningful may be more likely to overcome the cognitive and emotional barriers associated with loneliness and seek help when needed. First, work meaningfulness can improve attitudes toward help-seeking by reframing it as a responsible and purposeful action rather than a socially risky one. While lonely employees may otherwise view interpersonal engagement as exposing their vulnerability, those who find meaning in their work are more likely to see help-seeking as a necessary step to ensure the quality and success of their work (May et al., 2004). From COR standpoint, this positive reappraisal of help-seeking allows them to shift from a resource-conserving mindset toward a resource-gaining orientation. Help-seeking is thus seen not as a cost but as an intentional action that supports goal attainment under resource-depleted conditions.

Second, work meaningfulness can strengthen subjective norms by linking individual actions to a broader sense of collective responsibility. Employees who perceive their work as contributing to a greater good may view help-seeking not merely as acceptable, but as socially expected. This perception helps lonely employees overcome concerns about being negatively judged for seeking help (Cleavenger et al., 2007).

Third, meaningful work enhances perceived behavioral control by reinforcing employees’ belief that they can take effective action. When individuals see their work as important, they are more likely to feel responsible for outcomes and confident in managing challenges (M. H. Anderson, 2008; Hong & Gajendran, 2018). For example, a lonely employee might typically hesitate to ask for help, but the belief that their work matters may lead them to think, “I can handle this—I just need the right support.” This sense of control can empower employees to act despite the emotional withdrawal and self-doubt that often come with loneliness (Thomas & Velthouse, 1990).

**Hypothesis** **3.** *The negative relationship between workplace loneliness and help-seeking behavior is moderated by work meaningfulness, such that this relationship is attenuated when work meaningfulness is high but becomes stronger when work meaningfulness is low.*

## 3. Method

### 3.1. Sample and Procedure

We collected data from full-time employees in South Korea using an online survey administered by a professional survey panel provider. The survey was conducted over approximately one week in February 2025. Prior to data collection, we obtained ethical approval from the Institutional Review Board at Konkuk University (IRB protocol number: KKUIRB-202410-HR-008), and all participants provided informed consent in accordance with IBR guidelines. The original survey items were developed in English and were translated into Korean following standard back-translation procedures (Brislin, 1980), with two bilingual PhD students to ensure semantic equivalence. Before launching the full survey, four graduate students working in HR-related roles reviewed the translated items for clarity and relevance.

A total of 260 respondents participated in the survey. The sample was gender-balanced, with 130 men (50.0%) and 130 women (50.0%). Participants ranged in age from 23 to 59 years (M = 40.10, SD = 10.24). The average tenure at their current organization was 7.96 years (SD = 7.44). Educational attainment was measured on a 5-point ordinal scale and distributed as follows: 14.2% had completed high school or less (*n* = 37), 17.3% held a 2-year college degree (*n* = 45), 58.8% held a 4-year university degree (*n* = 153), 9.2% held a master’s degree (*n* = 24), and 0.4% held a doctoral degree (*n* = 1).

### 3.2. Measures

#### 3.2.1. Help-Seeking Behavior

We defined help-seeking behavior as an interpersonal action in which employees voluntarily request assistance from colleagues or supervisors to resolve problems or acquire resources necessary for task performance (S. E. Anderson & Williams, 1996). To assess this construct, we adapted five items originally developed by S. E. Anderson and Williams (1996), modifying them to reflect employees’ own help-seeking behavior rather than their perceptions of coworkers’ behavior. The items were translated into Korean and reviewed by two HR managers to ensure clarity, contextual relevance, and face validity. Responses were measured on a 5-point Likert scale (1 = strongly disagree to 5 = strongly agree). Example items include: “I ask my coworkers for help when I face difficulties at work,” “I am willing to seek assistance from my supervisor if I encounter problems I can’t solve alone,” and “When I need support to complete a task, I actively request it from others.” Higher scores indicated more frequent help-seeking behavior at work. The internal consistency of the scale was high (Cronbach’s α = 0.87).

#### 3.2.2. Workplace Loneliness

We measured workplace loneliness as a distressing emotional state that arises from a discrepancy between the desired and actual quality of social relationships in the workplace. We used the 9-item Workplace Loneliness Scale developed by Wright et al. (2007), which was translated into Korean for the present study. Participants rated each item on a 5-point Likert scale ranging from 1 (strongly disagree) to 5 (strongly agree). Example items include: “I often feel abandoned by my co-workers when I am under pressure at work,” “I often feel alienated from my co-workers,” “I feel myself withdrawing from the people I work with,” and “I often feel emotionally distant from the people I work with.” Higher scores indicated greater levels of workplace loneliness. The scale demonstrated excellent internal consistency (Cronbach’s α = 0.94).

#### 3.2.3. Perceived Transactive Memory System (TMS)

We measured TMS as a shared cognitive framework for encoding, storing, and retrieving information about who knows what within a team. Although TMS is traditionally assessed at the team level, we measured it at the individual level to capture each participant’s subjective perception of their team’s TMS. This individual-level approach was theoretically appropriate given our focus on how perceived TMS moderates the relationship between workplace loneliness and help-seeking behavior. We used a 15-item scale developed by Lewis (2003), encompassing three subdimensions: specialization, credibility, and coordination (five items each). Participants rated items on a 5-point Likert scale (1 = strongly disagree to 5 = strongly agree). Example items include “Each team member has specialized knowledge of some aspect of our project,” “I trusted that other members’ knowledge about the project was credible,” and “Our team worked together in a well-coordinated fashion.” We averaged all 15 items to compute a composite TMS score, with higher scores indicating stronger perceived TMS. Internal consistency was excellent for the overall scale (Cronbach’s α = 0.91), and acceptable across subdimensions: specialization (α = 0.80), credibility (α = 0.87), and coordination (α = 0.85).

#### 3.2.4. Work Meaningfulness

We measured work meaningfulness as a psychological resource through which individuals perceive intrinsic value in their work and integrate it with their self-identity and life goals. To assess this construct, we used a 6-item scale developed by May et al. (2004), which was translated into Korean for the present study. Participants rated each item using a 5-point Likert scale (1 = strongly disagree to 5 = strongly agree). Example items include: “The work I do on this job is very important to me,” “My job activities are personally meaningful to me,” and “I feel that the work I do on my job is valuable.” Higher scores reflected a stronger sense of perceived meaningfulness at work. The scale demonstrated excellent internal consistency (Cronbach’s α = 0.93).

#### 3.2.5. Control Variables

We included several control variables that could influence help-seeking behavior. First, we controlled for gender because the perceived cost of help-seeking may differ depending on gender-related status dynamics in the workplace (Hong et al., 2020; Rosette et al., 2015). Second, we controlled for organizational tenure, measured as the number of years the participant had worked at their current organization, given that help-seeking behaviors may vary depending on one’s level of organizational experience. We also controlled for educational attainment as a proxy for human capital. Education was coded on a 5-point ordinal scale (1 = less than high school, 2 = 2-year college, 3 = 4-year university, 4 = master’s degree, 5 = doctoral degree). We also controlled for self-rated job performance, as the degree to which individuals perceive themselves as performing well may influence their willingness to seek help (van der Vegt et al., 2006). Performance was measured using a 7-item in-role behavior scale developed by Williams and Anderson (1991) with a 5-point Likert scale. Example items include: “Adequately completes assigned duties,” “Fulfills responsibilities specified in job description,” and “Meets formal performance requirements of the job”(Cronbach’s α = 0.89).

## 4. Results

We conducted confirmatory factor analysis (CFA) to evaluate the construct validity of the measurement model. The hypothesized four-factor model—comprising help-seeking behavior, workplace loneliness, work meaningfulness, and transactive memory system (TMS)—demonstrated superior fit compared to alternative models (χ^2^(203) = 367, CFI = 0.961, TLI = 0.956, RMSEA = 0.056, SRMR = 0.056), providing strong empirical support for the discriminant validity of the four latent constructs (see Table 1).

We present the means, standard deviations, and correlations for the variables shown in Table 2. Although we expected a negative association, workplace loneliness was not significantly related to help-seeking behavior (r = −0.03, *p* = 0.676). Meanwhile, both the transactive memory system (r = 0.34, *p* < 0.01) and work meaningfulness (r = 0.20, *p* < 0.01) were positively associated with help-seeking behavior, suggesting that employees who perceive their team as cognitively coordinated or view their work as meaningful may be more inclined to seek help when needed. Gender also showed a significant relationship (r = −0.13, *p* < 0.05), with women reporting lower levels of help-seeking than men.

We conducted hierarchical regression analyses to examine the effects of workplace loneliness on employees’ help-seeking behavior, as well as the moderating roles of perceived TMS and work meaningfulness (see Table 3). Control variables (gender, education level, current tenure, and self-rated performance) were entered in Model 1. In Model 2, the main predictor—workplace loneliness—was added. Model 3 introduced the two proposed moderators, perceived TMS and work meaningfulness, without interaction terms. Models 4 and 5 tested the hypothesized interaction effects of TMS and work meaningfulness, respectively, while Model 6 included both interaction terms simultaneously (full model).

Hypothesis 1 predicted that workplace loneliness would be negatively associated with employees’ help-seeking behavior. Contrary to our expectation, the effect of workplace loneliness was not statistically significant (Model 3; b = −0.02, n.s.), providing no support for Hypothesis 1.

**Figure 2:**
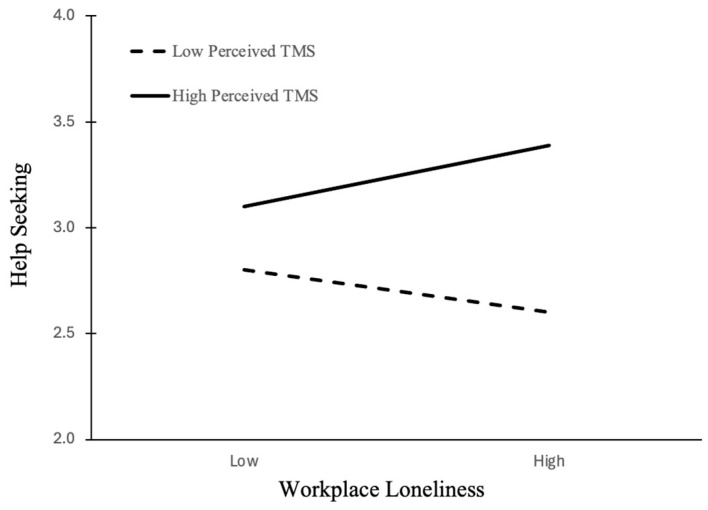
The moderation effect of perceived TMS on the relationship between workplace loneliness and help-seeking behavior. Notes: Workplace loneliness was positively associated with help-seeking when perceived TMS was high (I SD above the mean), but not when TMS was low. This suggests that perceived TMS can mitigate the negative effects of loneliness by facilitating adaptive engagement for help-seeking.

Hypothesis 2 proposed that the negative relationship between workplace loneliness and help-seeking behavior would be moderated by the perceived TMS, such that the relationship would be attenuated or even reversed when perceived TMS is high. Supporting Hypothesis 2, the interaction between workplace loneliness and perceived TMS was positive and significant (Model 4; b = 0.30, *p* < 0.01). Figure 2 graphically presents the interaction between workplace loneliness and TMS at high and low levels (1 SD above and below the mean). As expected, workplace loneliness had a stronger, positive effect on help-seeking when perceived TMS was high (simple slope b = 0.18, *p* < 0.05), but was not significant when TMS was low (simple slope b = −0.11, n.s.; see Table 4). To further assess the strength of the moderation effect, we calculated Cohen’s *f*^2^ for the ΔR^2^ between Model 3 and Model 4. This yielded a value of 0.044, which represents a small effect size based on Cohen’s (1988) benchmarks, indicating the effect is non-trivial).

**Figure 3:**
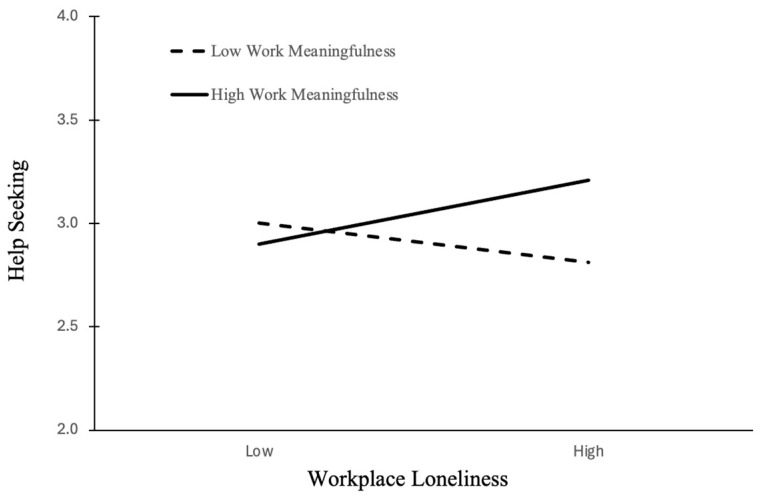
The moderation effect of work meaningfulness on the relationship between workplace loneliness and help-seeking behavior. Notes: Workplace loneliness was positively associated with help-seeking when work meaningfulness was high, but not when it was low. This indicates that work meaningfulness enables lonely employees to stay engaged rather than withdraw.

Hypothesis 3 predicted that the negative relationship between workplace loneliness and help-seeking behavior would be moderated by work meaningfulness, such that the relationship would be weakened or reversed when work meaningfulness is high. Supporting Hypothesis 3, the interaction between workplace loneliness and work meaningfulness was also positive and significant (Model 5; b = 0.23, *p* < 0.01). Figure 3 illustrates this interaction, showing that workplace loneliness was positively associated with help-seeking when work meaningfulness was high (simple slope b = 0.19, *p* < 0.05; see Table 5). Cohen’s *f*^2^ for the ΔR^2^ between Model 3 and Model 5 was 0.038, indicating a small effect size.

## 5. Discussion

### 5.1. Overall Findings

A central question in understanding help-seeking behavior within organizations is: When does workplace loneliness deter employees from reaching out to others, and under what conditions can this negative effect be mitigated or even reversed? The present study offers novel insights into these questions by integrating workplace loneliness with two key contextual moderators—perceived TMS and work meaningfulness—that shape employees’ willingness to seek help.

Our findings revealed that workplace loneliness alone was not significantly associated with help-seeking behavior, indicating that loneliness may not inherently discourage employees from reaching out to others. Instead, its impact appears to depend on specific contextual conditions. As hypothesized, perceived TMS moderated the relationship between loneliness and help-seeking, such that lonely individuals were more likely to seek help when they perceived high levels of shared cognitive structures in their team. Likewise, work meaningfulness emerged as a buffer, enabling lonely individuals to remain engaged in help-seeking when they perceived their work to be important and purposeful.

### 5.2. Theoretical Implication

This study makes several important theoretical contributions to research on workplace loneliness and help-seeking behavior by integrating insights from COR theory and the TPB.

First, we challenge the prevailing assumption that workplace loneliness uniformly leads to withdrawal and disengagement. COR theory suggests that lonely employees adopt a resource-protection orientation to avoid further depletion, and prior research has predominantly focused on how loneliness triggers avoidant behaviors such as reduced collaboration and social withdrawal (Ozcelik & Barsade, 2018; Xin et al., 2024). This leaves an important gap: under what conditions might lonely employees shift from resource protection to resource expansion? By integrating TPB, our study addresses this gap by demonstrating that workplace loneliness can have a negative effect on help-seeking, but this relationship is contingent on contextual factors that alter the lonely employees’ perceptions of control, normative appropriateness, and expected value—the psychological mechanisms central to TPB. Importantly, this does not imply that loneliness is beneficial; rather, it suggests that supportive workplace conditions can mitigate its detrimental effects by enabling lonely employees to engage in help-seeking behavior despite their relational deficits (J. T. Cacioppo et al., 2014; Gabriel et al., 2021; Qualter et al., 2015). This contributes to recent calls for research on when loneliness may lead to adaptive rather than purely avoidant outcomes in the workplace (McCarthy et al., 2025).

Second, by integrating TPB with COR theory, our study identifies two critical workplace conditions that shape whether loneliness leads to adaptive or avoidant behavior. Specifically, we propose that TMS and work meaningfulness serve as critical boundary conditions that align with TPB’s core components. When lonely employees perceive that knowledge is well-distributed and accessible in the team, or when they experience their work as purposeful, they are more likely to interpret help-seeking as a feasible, normatively supported, and valuable action. Under these conditions, the anticipated benefits of resources acquisition outweigh the psychological costs, enabling lonely employees to engage in proactive help-seeking. This integration advances our theoretical understanding of when and why resource-depleted employees choose resource-gaining behaviors over resource-conserving ones (J. T. Cacioppo et al., 2014; Gabriel et al., 2021).

Lastly, we extend the help-seeking literature by introducing workplace loneliness as a theoretically meaningful antecedent. While prior research has emphasized that help-seeking emerge when employees experience high relational quality (Bowler & Brass, 2006), trust their colleagues (Hofmann et al., 2009), or perceive reciprocity norms (Deckop et al., 2003). Our findings challenge this relational-strength paradigm by demonstrating that even employees experiencing relational deficits may engage in help-seeking behavior, particularly when enabling conditions are present. This suggests that help-seeking is not solely a function of existing relational capital but can also serve as a strategic response to relational scarcity—a means through which lonely employees attempt to acquire the social resources they lack. This perspective suggests help-seeking can be undermined by workplace loneliness but also used as a means to overcome it.

### 5.3. Practical Implication

Our model explained about 22% of the variance in help-seeking behavior, a level of explanatory power that is considered meaningful in micro-organizational research (Bosco et al., 2015). From a practical perspective, this suggests that the focal variables we identified (i.e., workplace loneliness, TMS, and work meaningfulness) capture a substantive and actionable portion of what drives help-seeking at work. Notably, while loneliness is often viewed as a problem to be alleviated, our findings indicate that under certain conditions, it can serve as a catalyst for constructive engagement, transforming loneliness into a potential driver of proactive action.

First, our findings highlight the importance of helping employees develop a clear perception of their team’s TMS. When individuals perceive that they know who holds what knowledge and how to access it, the social cost and uncertainty of seeking help are reduced. To support this, team leaders and HR practitioners should focus on making expertise within the team more visible and accessible through structured onboarding, group training, and feedback on team members’ strengths (Moreland & Myaskovsky, 2000), but these alone are not sufficient. TMS is not merely a static map of knowledge. Rather it is a dynamic system that evolves over time through ongoing collaboration, shared experience, and trust-building, which together strengthen accurate expertise recognition and coordination (Lewis, 2004; Majchrzak et al., 2012). These cumulative experiences are particularly important for lonely employees, as they help reduce the tendency to overestimate the risk of rejection and underestimate others’ willingness to help that often discourage support-seeking behavior (J. T. Cacioppo & Hawkley, 2009; Flynn & Lake, 2008; Qualter et al., 2015).

Furthermore, these efforts can be reinforced through broader organizational knowledge management strategies that amplify TMS development and facilitate help-seeking. For instance, implementing expert locator systems within the company intranet allows employees to find relevant knowledge holders beyond their immediate team, enhancing their ability to identify reliable sources (Choi et al., 2010). When these structures are in place, lonely employees are less likely to interpret help-seeking as exposing vulnerability and more likely to view it as a productive, normatively supported behaviors.

Finally, enhancing employees’ sense of work meaningfulness can serve as a motivational lever in encouraging help-seeking, particularly for those experiencing loneliness. As discussed earlier, meaningful work helps reframe help-seeking as a proactive and purpose-driven step toward achieving valued goals, rather than something to be avoided due to social discomfort. To support this, managers can create environments where employees feel their work contributes to a larger purpose. For example, providing regular feedback on impact, publicly recognizing contributions, and linking everyday tasks to a broader mission (e.g., reminding hospital staff that their work directly contributes to patient recovery) can help even routine work feel more purposeful and significant (Wrzesniewski et al., 2003; Rosso et al., 2010). To enhance work meaningfulness, managers can also provide autonomy support, giving employees greater control over how, when, and where they complete their work. When employees experience greater ownership over their work, they are more likely to seek help when needed to complete their responsibilities.

### 5.4. Limitation and Future Research

This study offers important contributions by empirically examining the relationship between workplace loneliness and help-seeking behavior and identifying TMS and work meaningfulness as key moderators. Nonetheless, several limitations should be acknowledged to guide future research. First, contrary to hypothesis 1, the direct association between workplace loneliness and help-seeking was not significant. We speculate that this null pattern may reflect cultural boundary conditions in our South Korean collectivist setting that lowering the perceived social costs of disclosure and activating a social-reconnection motive (Cho & Yoon, 2001). This motive could counterbalance the resource-protection tendency that loneliness typically triggers (e.g., withdrawal to avoid social risk; Fan et al., 2025), yielding a net effect near zero at the mean level. Looking ahead, specifying culturally boundary conditions would enhance the explanatory power and cross-cultural generalizability of the loneliness-help-seeking link.

Second, our methodological design also warrants caution: all key variables—loneliness, help-seeking behavior, and the proposed moderators—were assessed through self-reports at a single point in time, raising concerns about common method bias and limiting causal inference (Podsakoff et al., 2003). Future research would benefit from longitudinal and multi-source designs. For example, help-seeking behavior could be assessed through peer or supervisor ratings, and TMS could be measured as a team-level construct by aggregating responses across team members (e.g., Lewis, 2003), allowing for more robust operationalization of key constructs and minimizing the influence of self-reporting bias.

Third, our study provides a workplace-focused understanding of loneliness, centering on the proximal resource context for workplace behaviors. While this targeted approach is theoretically grounded, it does not capture the potential buffering role of non-work social resources (e.g., support from family or close friends) which may compensate for resource depletion experienced at work (Greenhaus & Powell, 2006)). Future research could examine whether such cross-domain substitution effects moderate the behavioral consequences of workplace loneliness.

Moreover, our study treated workplace loneliness as a unidimensional construct, consistent with prior organizational research. Yet emerging evidence suggests that workplace loneliness is multidimensional, including social loneliness (feeling a lack of belonging to a group) and emotional loneliness (lacking intimate one---on---one connections), as well as distinctions between chronic versus situational loneliness (S. Cacioppo et al., 2015). These different forms may carry distinct implications for behavior and motivation. In addition, even an individual’s general level of loneliness may fluctuate depending on specific work situations, such as the type of task being performed (e.g., collaborative vs. solo) or even the day of the week. For example, chronic emotional loneliness might be more strongly impacted by work meaningfulness—an enduring sense of purpose—whereas situational social loneliness might be more responsive to team-level structures like TMS, which guide day---to---day interactions. Future studies should disaggregate these dimensions to examine whether certain types of loneliness trigger different helping behaviors or respond to different enabling conditions.

Finally, we acknowledge that a significant portion of variance in help-seeking behavior remains unexplained. This underscores the complex, multi-determined nature of help-seeking and highlights the importance of exploring additional explanatory variables. For example, other contextual moderators may play critical roles. Informed by the TPB, factors such as team climate, leadership style, and organizational norms may shape employees’ attitudes, perceived behavioral control, and normative support for seeking help. Exploring these broader range of contextual enablers will deepen our understanding of when and how workplace loneliness leads to adaptive engagement like help-seeking rather than avoidance or withdrawal, moving the literature toward a more nuanced, context-sensitive model of loneliness within organizational life (McCarthy et al., 2025).

## 6. Conclusions

Loneliness has increasingly been recognized as a widespread social and health concern, and workplaces are no exception. Often regarded primarily as a negative experience, loneliness can in fact be a natural human experience. The critical question, therefore, is how to turn the workplace loneliness into actions that rebuild social ties and promote cooperation. This study addresses that question by examining when workplace loneliness leads to positive outcomes—in particular, help-seeking among South Korean employees.

Our findings contribute to theory by showing that loneliness does not invariably inhibit interpersonal engagement; under certain contextual (perceived transactive memory systems) and motivational (work meaningfulness) conditions, it can motivate employees to seek assistance. This challenges views of loneliness that focus only on its downsides and aligns with emerging perspectives on its potential adaptive functions.

Practically, the results suggest that organizations should move beyond simply trying to reduce loneliness and instead create conditions that encourage lonely employees to engage in help-seeking. Strengthening employees’ awareness of who holds what expertise can not only lower the perceived social costs of asking for help but also foster positive attitudes toward seeking assistance, strengthen the perception that it is socially supported, and enhance confidence in one’s ability to do so. Likewise, fostering work meaningfulness can help employees see help-seeking as a purposeful step toward achieving important goals rather than as a sign of weakness. By shaping these contextual and motivational factors, organizations can enable lonely employees to turn their experience into proactive help-seeking that benefits both individuals and teams.

## Figures and Tables

**Table 1:** Model Fit Indices for Confirmatory Factor Analyses.

Model Structure	*χ*^2^ (*df*)	CFI	TLI	SRMR	RMSEA
4-Factor Model: Seeking, Loneliness, Meaning, TMS	367 (203)	0.961	0.956	0.056	0.056
3-Factor: Seeking, Loneliness, Meaning + TMS	604 (206)	0.906	0.895	0.081	0.086
2-Factor: Seeking, Loneliness + Meaning + TMS	1795 (208)	0.625	0.584	0.198	0.171
1-Factor: All Items on Single Factor	2380 (209)	0.487	0.433	0.224	0.200

Notes: The hypothesized 4-factor model (help-seeking, workplace loneliness, work meaningfulness, TMS) showed superior fit compared to all alternative models, with CFI and TLI exceeding 0.95, and RMSEA and SRMR below 0.06. This supports the discriminant validity of the four constructs used in the study.

**Table 2:** Means, standard deviation, and correlations between the study variables.

	Mean	S.D.	(1)	(2)	(3)	(4)	(5)	(6)	(7)
(1) Gender	0.50	0.50	-						
(2) Education Level	2.64	0.85	−0.09	-					
(3) Current Tenure (years)	7.96	7.44	−0.01	−0.05	-				
(4) Self-rated Job Performance	3.74	0.52	−0.03	0.17 **	0.10	-			
(5) Perceived TMS	3.54	0.49	−0.03	0.18 **	0.04	0.44 **	-		
(6) Work Meaningfulness	3.60	0.69	−0.04	0.05	0.05	0.39 **	0.44 **	-	
(7) Workplace Loneliness	2.38	0.82	−0.01	−0.06	−0.02	−0.22 **	−0.22 **	−0.25 **	-
(8) Help-Seeking	2.95	0.78	−0.13 *	−0.10	−0.09	0.02	0.34 **	0.20 **	−0.03

Note: N = 260. * *p* < 0.05. ** *p* < 0.01. Two-tailed test. Gender was coded as 0 = male, 1 = female.

**Table 3:** Results of the hierarchical regression analysis.

	Model 1		Model 2		Model 3		Model 4		Model 5		Model 6	
	Coeff. (S.E.)	95% C.I. [LL, UL]	Coeff. (S.E.)	95% C.I. [LL, UL]	Coeff. (S.E.)	95% C.I. [LL, UL]	Coeff. (S.E.)	95% C.I. [LL, UL]	Coeff. (S.E.)	95% C.I. [LL, UL]	Coeff. (S.E.)	95% C.I. [LL, UL]
Intercept	2.95 ** (0.05)	[2.85, 3.04]	2.95 ** (0.05)	[2.85, 3.04]	2.95 ** (0.04)	[2.86, 3.04]	2.97 ** (0.04)	[2.88, 3.06]	2.98 ** (0.05)	[2.89, 3.07]	2.99 ** (0.04)	[2.90, 3.08]
Gender	−0.20 * (0.10)	[−0.39, −0.01]	−0.20 * (0.10)	[−0.39, −0.01]	−0.19 * (0.09)	[−0.36, −0.01]	−0.20 * (0.09)	[−0.38, −0.03]	−0.22 * (0.09)	[−0.40, −0.05]	−0.22 * (0.09)	[−0.40, −0.05]
Education level	0.08 (0.06)	[−0.03, 0.19]	0.08 (0.06)	[−0.04, 0.19]	0.05 (0.05)	[−0.06, 0.15]	0.05 (0.05)	[−0.06, 0.15]	0.04 (0.05)	[−0.07, 0.14]	0.04 (0.05)	[−0.06, 0.14]
Current tenure (years)	−0.01 (0.01)	[−0.02, 0.00]	−0.01 (0.01)	[−0.02, 0.00]	−0.01 (0.01)	[−0.02, 0.00]	−0.01 (0.01)	[−0.02, 0.00]	−0.01 (0.01)	[−0.02, 0.00]	−0.01 (0.01)	[−0.02, 0.00]
Self-rated Performance	0.01 (0.09)	[−0.17, 0.20]	0.01 ( 0.10)	[−0.19, 0.19]	−0.28 ** (0.10)	[−0.48, −0.08]	−0.27 ** (0.10)	[−0.47, −0.08]	−0.27 ** (0.10)	[−0.47, −0.08]	−0.27 ** (0.10)	[−0.46, −0.08]
Workplace Loneliness			−0.02 (0.06)	[−0.14, 0.10]	0.04 (0.06)	[−0.07, 0.15]	0.03 (0.06)	[−0.08, 0.14]	0.03 (0.06)	[−0.08, 0.15]	0.03 (0.06)	[−0.08, 0.14]
Perceived TMS					0.61 ** (0.11)	[0.39, 0.82]	0.56 ** (0.11)	[0.34, 0.77]	0.58 ** (0.11)	[0.37, 0.79]	0.55 ** (0.11)	[0.34, 0.77]
Work Meaningfulness					0.13 (0.08)	[−0.02, 0.28]	0.12 (0.07)	[−0.03, 0.26]	0.11 (0.08)	[−0.04, 0.26]	0.11 (0.07)	[−0.04, 0.25]
Workplace Loneliness × TMS							0.30 ** (0.09)	[0.12, 0.47]			0.22 ** (0.10)	[0.04, 0.41]
Workplace Loneliness × Work Meaningfulness									0.23 ** (0.07)	[0.08, 0.37]	0.15 * (0.07)	[0.00, 0.31]
R^2^	0.03		0.03		0.18		0.21		0.21		0.22	
ΔR^2^			0.00		0.14 **		0.04 **		0.03 **		0.05 **	

Note: N = 260. * *p* < 0.05. ** *p* < 0.01. Two-tailed test. R^2^ change values in Models 4, 5, and 6 are calculated based on Model 3 as the reference model. All values are rounded to the nearest third decimal place. Changes in explained variance for Models 4, 5, and 6 were calculated relative to Model 3. The interaction terms in Models 4, 5, and 6 significantly increased the explained variance in help-seeking behavior, supporting the hypothesized moderation effects of perceived TMS and work meaningfulness.

**Table 4:** Simple slope test of perceived TMS for help-seeking behavior.

	Slope	S.E.
Mean − 1 SD	−0.11	0.07
Mean + 1 SD	0.18 *	0.07

Notes: Simple effects are estimated, keeping other independent variables constant in the model. The positive relationship between workplace loneliness and help-seeking was observed only when perceived TMS was high (1 SD above the mean); the association was not significant when TMS was low. * *p* < 0.05.

**Table 5:** Simple slope test of work meaningfulness for help-seeking behavior.

	Slope	S.E.
Mean − 1 SD	−0.12	0.08
Mean + 1 SD	0.19 *	0.07

Note: Simple effects are estimated, keeping other independent variables constant in the model. Workplace loneliness was positively associated with help-seeking only at high levels of work meaningfulness; no significant association was observed when work meaningfulness was low. * *p* < 0.05.

## Data Availability

The data that support the findings of this study are available from the corresponding author upon reasonable request.

## Abbreviations

The following abbreviations are used in this manuscript:

COR	Conservation of Resources
TMS	Transactive Memory System
TPB	Theory of Planned Behavior
